# Electrochemically primed functional redox mediator generator from the decomposition of solid state electrolyte

**DOI:** 10.1038/s41467-019-09638-4

**Published:** 2019-04-23

**Authors:** Matthew Li, Zhengyu Bai, Yejing Li, Lu Ma, Alvin Dai, Xuefeng Wang, Dan Luo, Tianpin Wu, Ping Liu, Lin Yang, Khalil Amine, Zhongwei Chen, Jun Lu

**Affiliations:** 1 School of Chemistry and Chemical Engineering, Key Laboratory of Green Chemical Media and Reactions, Ministry of Education, Henan Normal University, 453007 Xinxiang, China; 2 Department of Chemical Engineering, Waterloo Institute of Nanotechnology, University of Waterloo, 200 University Avenue West, Waterloo, ON N2L 3G1 Canada; 3 Chemical Sciences and Engineering Division, Argonne National Laboratory, 9700 Cass Avenue, Lemont, IL 60439 USA; 4 Department of NanoEngineering, University of California San Diego, 9500 Gilman Drive, La Jolla, CA 92093 USA; 5 X-ray Science Division, Advanced Photon Source, Argonne National Laboratory, 9700 Cass Avenue, Lemont, IL 60439 USA; 6 Department of Macromolecular and Science and Engineering, School of Engineering, Case Western Reserve University, 2100 Adelbert Road, Cleveland, OH 44106 USA

**Keywords:** Batteries, Batteries, Electrochemistry

## Abstract

Recent works into sulfide-type solid electrolyte materials have attracted much attention among the battery community. Specifically, the oxidative decomposition of phosphorus and sulfur based solid state electrolyte has been considered one of the main hurdles towards practical application. Here we demonstrate that this phenomenon can be leveraged when lithium thiophosphate is applied as an electrochemically “switched-on” functional redox mediator-generator for the activation of commercial bulk lithium sulfide at up to 70 wt.% lithium sulfide electrode content. X-ray adsorption near-edge spectroscopy coupled with electrochemical impedance spectroscopy and Raman indicate a catalytic effect of generated redox mediators on the first charge of lithium sulfide. In contrast to pre-solvated redox mediator species, this design decouples the lithium sulfide activation process from the constraints of low electrolyte content cell operation stemming from pre-solvated redox mediators. Reasonable performance is demonstrated at strict testing conditions.

## Abstract (web summary)

The decomposition of solid state electrolyte material has been well-known in the literature. Here the authors report that the same decomposition process can be leveraged to act as a source of redox mediator that is only activated at certain voltages for application in Li_2_S based cathodes.

## Introduction

The class of sulfide-type solid electrolyte (STSSE) materials has gained significant interest among the solid-state electrolyte community^1–4^. Their tendency to possess higher Li-ion conduction and possibilities of low temperature synthesis (when compared to the next leading solid electrolyte material)^5^ makes them one of the most promising candidate for solid-state batteries^6^. Recent research into the STSSE has placed a spotlight on the electrochemical stability of STSEE. Thermodynamically, STSSEs are known to be unstable at the voltage range of interest for both cathode and anode electrodes^7,8^. Specifically, the nature of the interface layer (i.e., the degree and type of passivation) between the STSSE and active material strongly dictates the longevity of a solid-state cell^9,10^. With so much knowledge gained on the decomposition process of STSSE, it is timely to examine cross-field applications of this phenomenon. Some of the thermodynamically predicted oxidation decomposition products (particularly in the case of redox-active products^8,11,12^) of P- and S-containing STSSE can be quite useful for liquid battery systems where redox mediators are needed^13^. Furthermore, as these products are only produced when the STSSE is oxidized, it can present itself as an in situ generator of chemical species that can be “switched-on” at specific oxidation voltages.

A highly applicable area for these functional redox mediators is the sulfur-based battery chemistries^14,15^. As a promising cathode material for Li-S or S-based Li-ion batteries, Li_2_S can not only supply a source of Li ions but can also mitigate the volume expansion concerns of the sulfur electrodes. With a specific Li-ion capacity of over 1100 mAh g^−1^, Li_2_S has drawn much recent attention in the research community. While the benefits are clear^16^, Li_2_S presents a very high initial activation barrier for the first charge, which stems from the strong ionic bonds within its crystal and the nucleation barrier of forming solvated polysulfide species^17^. Such a hurdle (often reaching >4.2 V vs. Li^+^/Li) typically results in low specific capacity and electrolyte decomposition leading to poor cycle stability. Strategies used to solve these problems typically fall into two categories: carefully designed nano-sized Li_2_S composite material^18,19^ or electrolyte additive in the form of pre-solvated redox mediators^17,20^. While both of these strategies have shown significant progress in reducing the charge overpotential and achieving good overall performance, they also introduce significant disadvantages. Novel Li_2_S composite materials typically require exotic synthesis environments composing of high temperatures or toxicity, while soluble redox mediators often introduced into the electrolyte will contribute to severe internal shuttling and inevitably depend on the volume of electrolyte used. Furthermore, the use of pre-solvated redox mediators will likely deteriorate in effectiveness if the cells are not immediately charged after assembly, conflicting with the electrolyte wetting process. Moreover, the commonly used excess amounts of high oxidation state polysulfides as redox mediators will result in an overall low amount of starting Li ion from the cathode (for a full cell Li_2_S-based lithium ion battery (LIB)). An ideal solution to these challenges is the use of an initially dormant redox mediator that remains inactive during the electrolyte wetting process, effective at low mass ratios, separate from the electrolyte to decouple from the effect of electrolyte volume, and only activates when the first charge is initiated.

In this work, we demonstrate that the oxidative decomposition of solid Li_3_PS_4_ (lithium thiophosphate (LPS)) can be leveraged as a redox mediator generator that is electrochemically “switched-on” for lowering the first charge overpotential of commercial Li_2_S. Crucially, the observed enhancements in performance are achieved with only the direct simple mixing of LPS into the slurry formulation at 10 wt.%. This is the first reported use of a solid material as a redox mediator source. We have shown that this material will not dissolve into the electrolyte unless activated, and as such, less dependent on the volume of electrolyte used.

## Results

### Disadvantage of pre-solvated redox mediator: lithium polysulfide

Electrolytes preloaded with soluble redox mediators have been widely used as Li_2_S activators throughout literature^17,20,21^. Taking lithium polysulfide (LiPS) as the representative additive, it is clear that the use of Li_2_S_8_ as redox mediator is only applicable when significant amounts of Li_2_S_8_ are added (Supplementary Figure 1a, arrows indicates the theoretical amount of Li ion that is extractable from each specific ratio of Li_2_S to Li_2_S_8_). For an electrode based on a 60 wt.% commercial Li_2_S, it was found that the ratio of Li_2_S_8_ to Li_2_S must be raised to a relatively high (30–60 wt%) to achieve significant improvements to the charge voltage profile. It should be noted that this is not the result of a decreased current density (normalized to loaded Li_2_S mass) as experiments at half the current still maintains a very high charge voltage (Supplementary Figure 1b). It is important to remember that one of the most advantageous aspect of using Li_2_S over S is its ability to serve as a source of Li ions, that is, pairing with a Li-ion-free anode. It is then foreseeable that the use of significant amounts of LiPS to activate Li_2_S will result in a delithiated cathode with excess amounts of sulfur that cannot be lithiated in the subsequent cycle without another source of Li-ions. Additionally, every sample with soluble polysulfide species experienced higher than theoretical charge specific capacity (theoretical indicated by arrowheads) likely suggesting shuttling^22^. This is particularly evident in cells (LiPS:Li_2_S = 30:70) cycled at decreased current density (Supplementary Figure 1c), yielding a first charge capacity of well over 2500 mAh g^−1^ normalized to the sulfur content even with 0.5 M LiNO_3_. Finally, changes to the electrolyte content also dramatically influences the chargeability of the electrode. When the total electrolyte content is tuned down to ~$7\,{\rm{\mu}}{\mathrm{{L}}}\,{\mathrm{mg}}^{-1}_{{{\rm{{Li}}}_{2}}{\rm{S}}}$ (maintaining a constant LiPS:Li_2_S ratio), there is a dramatic decrease in specific capacity and corresponding increase in charge potential. Therefore, a Li_2_S-based LIB that utilizes polysulfide in amounts such that it serves as an efficient Li_2_S activator and under low electrolyte conditions, will be practically unlikely.

### Electrochemical properties of LPS and its application in commercial Li_2_S cathodes

**Fig. 1:**
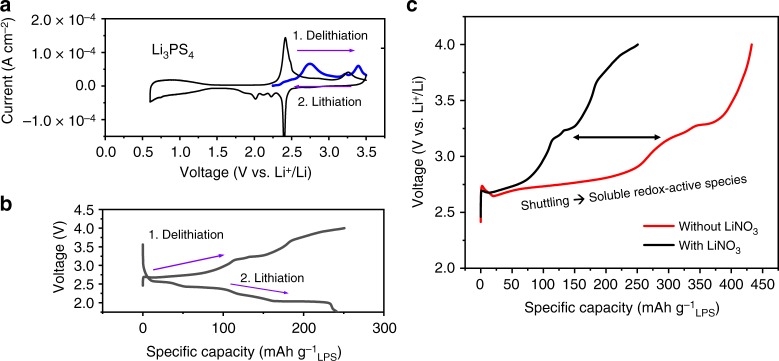
Electrochemical data of lithium thiophosphate (LPS) cycled in liquid ether-based electrolyte. **a** Cyclic voltammetry of LPS with an initial anodic sweep (blue) and subsequent cycling in black, **b** galvanostatic charge/discharge of LPS with LiNO_3_ in electrolyte, and **c** first charge of LPS with and without LiNO_3_ in electrolyte suggesting shuttling

The specific LPS solid electrolyte composition has drawn a lot of interest due to its high ionic conductivity at room temperature and ease of synthesis^2,23^. The stability of LPS has been a topic of great concern in the field of solid electrolyte^24–26^. It is often considered that the electrochemical decomposition of LPS complicates its application as a solid-state Li-ion conductor. Due to its redox activity at a potential window similar to that of Li_2_S^8^, it would be interesting to revisit the stability of LPS, but in a liquid electrolyte setting. With a similar but slightly higher oxidation voltage than Li_2_S, it could be a perfect source of redox mediators for Li_2_S activation. Figure 1a displays the cyclic voltammograms of LPS in common 1,3 dioxolane/dimethoxymethane-based Li-S electrolyte. A pronounced anodic peak is found at ~2.7 V and then at ~3.4 V during its initial delithiation sweep (in blue). The following cathodic and subsequent anodic sweep (in black) further demonstrated its redox activity in Li-S electrolyte. Corroborating these data, constant current delithiation of LPS at 0.1 mA mg^−1^ produces a sloped plateau from ~2.7 to 3.1 V (Fig. 1b) followed by another plateau at ~3.2 and 3.6–4.0 V. While these results are intriguing and produced apparently more pronounced voltage profiles from traditional solid-states studies of LPS^27^, the most important phenomenon of LPS can only be revealed when charged without LiNO_3_ present in the electrolyte. Cells charged with LiNO_3_ yielded ~250 mAh g^−1^_LPS_, whereas cells without LiNO_3_ (commonly used to prevent shuttling^28^) required more than 430 mAh g^−1^ (Fig. 1c) with a prolonged ~2.7–3.1 V sloped plateau. Furthermore, post cycling imaging (scanning electron microscopy, Supplementary Figure 2a) and elemental analysis (energy-dispersive spectroscopy, Supplementary Figure 2b) of the cycled Li metal counter electrode revealed a surface layer composed of both phosphorus and sulfur. Taken together, this provides strong evidence that the longer voltage plateau of the cell charged without LiNO_3_ is due to shuttling of an electrolyte soluble redox-active species that is generated by LPS upon oxidative decomposition.

**Fig. 2:**
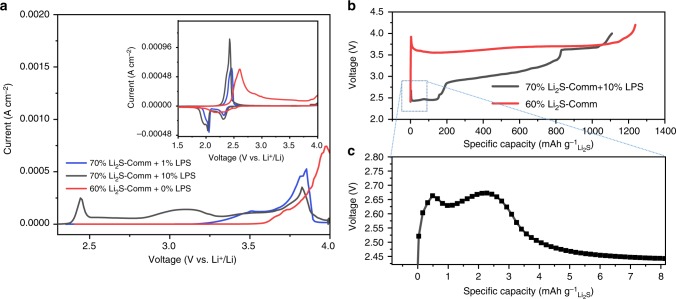
First charge electrochemical properties of lithium thiophosphate (LPS) blended into commercial Li_2_S (Li_2_S-Comm) as an electrochemically “switched-on” redox mediator generator. **a** Cyclic voltammetry of various combination of LPS and Li_2_S-Comm, inset shows the subsequent cathodic→anodic sweep cycle after the initial anodic activation sweep, and **b** first charge voltage profile of electrode using 10% LPS with 70% Li_2_S-Comm at C/20 with **c** magnified view showing double peak

With the generation of redox-active species starting at 2.7 V (close to, but higher than the oxidation potential of Li_2_S), LPS is an ideal candidate as a solid redox mediator generator that can be electrochemically “switched-on,” dissolving into the electrolyte in parallel to Li_2_S charging. We now demonstrate here that these decomposition products can be leveraged to enhance the electrochemical performance of cathodes based on even commercial micron-sized Li_2_S (Comm-Li_2_S) powder. By simply blending bulk LPS into the slurry formulation of Comm-Li_2_S electrode, significant enhancements in the electrochemical properties were achieved. Supplementary Figure 3a, b indicates that the Li_2_S is very much crystalline, producing pronounced peaks. Furthermore, the small peaks at the lower 2*θ* persisted even while submerged in the electrolyte (1 M lithium bis(trifluoromethanesulfonyl)imide (LiTFSI) + 0.5 LiNO_3_ in dimethoxyethane/1,3 dioxolane (DME/DOL) (1:1 v/v)), indicating that LPS does not dissolve into anhydrous electrolyte formulations. However, it should be noted that the crystal structure of Li_3_PS_4_ changed after electrode fabrication as the X-ray powder diffraction peaks have changed. Figure 2a displays the cyclic voltammetry of fresh Comm-Li_2_S electrode prepared with 1 and 10 wt% LPS. Compared to the pure Comm-Li_2_S, the charge overpotential was observed to decrease with increasing LPS ratio. Even at a minimal LPS ratio of 1 wt %, an initial anodic peak was found at 3.5 V with a subsequent peak at 3.8 V, which is lower than the ~4.0 V required to obtain an oxidative peak for the pure Comm-Li_2_S electrode. At 0.05C, the charge voltage of Comm-Li_2_S was dramatically decreased (Fig. 2b) even with an increased Li_2_S content. It is also worth mentioning that there appears to be a double activation peak as shown in Fig. 2c. We believe the first is the initial “switching-on” process of LPS, followed by its activation (second peak) of the bulk Li_2_S assisted by the oxidation products of LPS. After the initial activation process, there is an initial plateau followed by a sloped second plateau and a third plateau at the higher voltage region. The segregation of these plateaus is likely related to the different activation processes of Li_2_S with LPS. For example, the initial plateau could be due to the higher concentration of redox mediators, while the second sloped plateau could be an indication of a gradual decline in redox mediator concentration.

### The role of LPS in Li_2_S activation

**Fig. 3:**
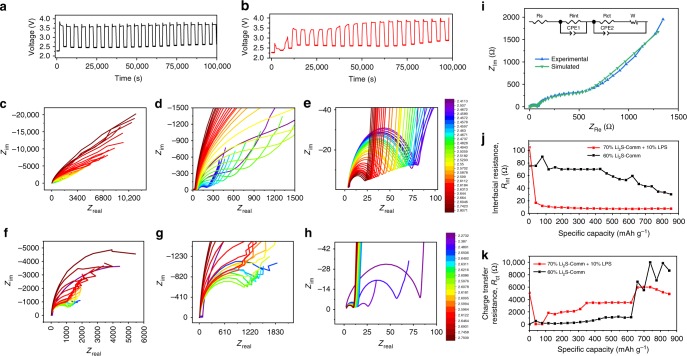
Electrochemical impedance spectroscopy (EIS) study. Voltage profile of EIS experiment of **a** the commercial Li_2_S (Li_2_S-Comm) electrode and **b** the 70% Li_2_S-Comm blended with 10% lithium thiophosphate (LPS) electrode. **c**–**e** Corresponding Nyquist plot of the Li_2_S-Comm electrode and **f**–**h** 70% Li_2_S-Comm blended with 10% LPS electrode at different axis ranges. The color legend is labeled in V. **i** Example of experimental and simulated Nyquist plot, **j** interfacial resistance (high-frequency semicircle) and **k** plot of charge transfer resistance (mid-frequency semicircle) of Comm-Li_2_S (black) and with LPS (red)

Electrochemical impedance spectroscopy (EIS) reveals substantial differences between electrode with and without LPS. Figure 3a, b shows the voltage profile during the operando EIS experiment of Comm-Li_2_S at 60% in black and Comm-Li_2_S at 70% with 10% LPS in red, respectively. It should be noted that the voltage profile of the EIS process varies from the pure galvanostatic charge process likely due to the dynamic nature of soluble species created. Although EIS processes have been considered mostly non-invasive in most studies, this result reveals a clear reduction in effectiveness in LPS’s activation of Li_2_S. Furthermore, there also appears to be an apparent requirement of the pure Comm-Li_2_S cells to reinitiate its activation process with a sharp peak after each EIS data collection session (Fig. 3a). This shows that the activation process of Li_2_S is very time dependent likely with concurrent processes competing for polysulfide (out-of-cathode diffusion and anode corrosion)^29^.

Regardless, important differences in impedance are still observed. The Nyquist plot of the collected EIS data of pure Li_2_S-Comm electrode is shown in Fig. 3c–e and of the 70% Li_2_S-Comm blended with 10% LPS electrode in Fig. 3f–h. As the impedance features of this material varies significantly with frequency, different axis range/magnifications are shown for clarity. Consistently throughout the first charge, a small semi-circle was found at the high-frequency range followed by a very large mid-frequency semi-circle, which we allocated to interfacial and charge transfer resistance, respectively, according to previous temperature-based studies^30^. Based on this, all Nyquist plots were fitted as shown in Fig. 2i (corresponding simulated and experimental Bode plot can be found in Supplementary Figure 4a, b, respectively). Each impedance spectrum was fitted to the circuit shown in inset of Fig. 2i^18,31–33^. Initially, the interfacial resistance appeared to be higher for the cells with LPS (Fig. 3j). This could be attributed to the higher ratio of non-conductive material (LPS and Li_2_S) to carbon as the Li_2_S content is 70% in the electrode containing LPS, whereas the electrode without LPS has only 60%. Interestingly, after the initial activation process, the interfacial resistance of the LPS-containing electrode dropped drastically from ~105 to ~10 Ω. This is in contrast to the pure 60% Li_2_S electrode where it exhibited only a modest decrease throughout the course of charge. Additionally, the charge transfer resistance (mid-frequency semi-circle) initially started higher once again for the LPS-containing electrode, but was exceeded by the pure Li_2_S electrode near the end of the charge period (~650 mAh g^−1^) as shown in Fig. 3k. This is intriguing because the enormous charge transfer resistance of the LPS-containing electrode is not reflected in the charging voltage profile. As the impedance spectroscopy was taken at potentiostatic conditions after a rest period (i.e., spectrum collection voltage is close to open-circuit voltage (OCV) at each specific state of charge), the resulting current response oscillates around near 0 mA. This suggests that there is a very strong dependence of the charge voltage on the presence of an applied current. It is further intriguing that for the pure Li_2_S electrode, after each rest period and subsequent EIS spectrum analysis, there is a reactivation process indicating a depletion of polysulfide from the previous current-halt period (rest time and EIS analysis). This is not present in the first ~400 mAh g^−1^ of charge for the electrodes with LPS. Accordingly, the contradiction between the higher charge voltage and lower apparent impedance of the LPS-containing electrode is likely due to good charge transfer kinetics of polysulfide and its role in the comproportionation reaction with Li_2_S^34^. However, because there is a constant need of the pure Comm-Li_2_S electrode to generate polysulfide species that are quickly consumed (by other polysulfide competing processes^29^). This creates a situation where the polysulfides cannot properly react with the remaining Li_2_S, and as such, the voltage remains high. Whereas in the case of the LPS-containing electrode, the lack of an activation process after each EIS analysis indicates significantly higher amounts of soluble mediators that are generated with longer lifetime, serving a more prolonged role in mediating the charge process of the bulk Li_2_S particles.

**Fig. 4:**
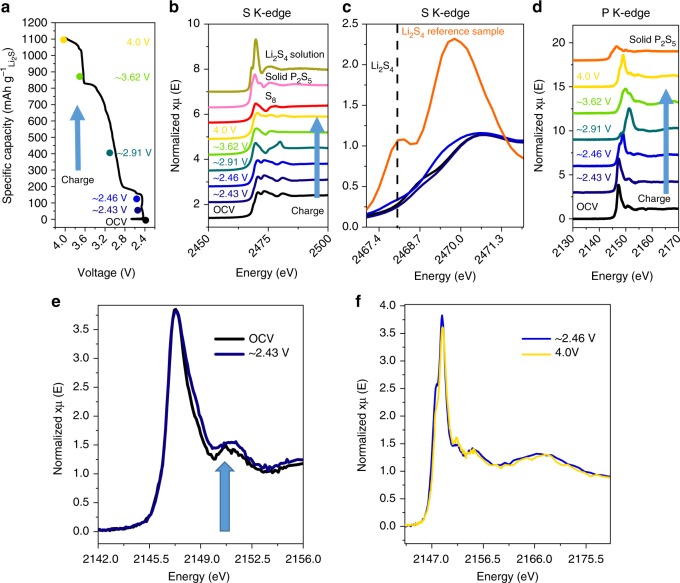
Ex situ X-ray adsorption near edge spectroscopy study of lithium thiophosphate (LPS) containing Li_2_S electrodes at various voltage throughout the first charge. **a** Voltage profile of the first charge (0.05C) of 70% commercial Li_2_S (Li_2_S-Comm) + 10% LPS, where the circles indicate the specific capacity/voltage at which each ex situ X-ray absorption spectroscopy (XAS) measurement was conducted. **b** S K-edge of electrode taken at different states of charge with spectra of homemade Li_2_S_4_ solution, commercial P_2_S_5_ and commercial S_8_ as a reference. **c** Magnified S K-edge of electrode opened at: fresh and ~2.46 V. **d** P K-edge of electrodes taken at different states of charged in addition to commercial P_2_S_5_ as reference. **e** Overlay of P K-edge at OCV and ~2.43, with the arrow indicating the increase in near edge features and **f** ~2.46 and 4.0 V displaying highly similar features at the beginning (~2.46 V) and end of charge (4.0 V)

To clarify the mechanism of LPS on the charging process of Li_2_S, ex situ X-ray absorption near-edge structure (XANES) was conducted. As indicated on Fig. 4a, six spectra were measured at six different states of charge. Specifically, cells were disassembled and analyzed at OCV = ~2.42, ~2.43, ~2.46, ~2.91, ~3.62, and 4.0 V. Overall, the S K-edge was found to change considerably over the course of the first charge (Fig. 4b). From OCV to ~2.46 V, the overall characteristics of the S K-edge remained relatively the same where the convex shape of the Li_2_S aligns well with the literature^35^. However, in the magnified view (Fig. 4c), it can be seen that the polysulfide shoulder (~2468 eV, matching our polysulfide standard measurement) increases from the fresh cell at OCV (Fig. 4c, black curve) to the cell at ~2.46 V (Fig. 4c, blue). Interestingly, the polysulfide species were not detected at the lower state of charge of ~2.43 V (Fig. 4c, dark blue), whereas a change in the P K-edge was detected early in the charge process (~2.43 V, Fig. 4d, e). This strongly suggests that the initial activation process of Li_2_S is not due to the formation of polysulfide^35^. At higher voltages (~3.62 V) the spectrum largely resembles that of the sulfur standard sample (Fig. 4b), where the convex shape of Li_2_S changes to concave at ~2474 eV as previously reported^35^. In fact, the convex shape of Li_2_S cannot be found even at ~2.91 V, suggesting that a large majority of it has been consumed, that is, likely successfully oxidized. This is also seen in the ex situ Raman spectroscopy (Supplementary Figure 5) of the same electrodes where the peak at ~365 cm^−1^ (likely Li_2_S) shifts to 354 cm^−1^ from ~2.43 to ~2.46 V whereupon it completely disappears at voltages above ~2.46 V. At 4.0 V, the spectrum further evolves into a shape different to that of the S_8_ standard sample. The origin of this pattern is however unclear, but we believe it might be some phosphorus-based sulfur species superimposed by elemental sulfur signals as theoretically predicted by the high voltage decomposition of Li_3_PS_4_^8^. Surprisingly, the P K-edge of the sample at 4.0 V did not exhibit features similar to the P_2_S_5_ standard (Fig. 4d). This is also seen in the Raman data where the originally formed ~246.5 cm^−1^ at the ~3.62 V transforms into a double peak centered at ~241 and 264 cm^−1^ at 4.0 V (Supplementary Figure 5), not present in the commercial orthorhombic sulfur sample^36^. It is worth noting that P_2_S_5_ is one of the thermodynamically predicted oxidation products of LPS at high voltage^8^.

It is important to note the difference between our XANES data and other previous reports on the delithiation of LPS in a solid-state configuration^27^. While the initial P K-edge features of our original OCV samples are near identical (Fig. 4e) to the literature, the XANES feature differed quite drastically upon delithiation in our work. In the solid-state configuration, the P K-edge was reported to remain mostly constant throughout delithiation with only decrease in the edge-peak height coupled with a general increase of the broad peaks at higher energy levels. Interestingly, an analogous process of decreasing the synthesis molar ratio of Li_2_S:P_2_S_5_ (LPS, molar equivalent of 75 Li_2_S:25 P_2_S_5_), that is, decreasing the proportion of Li, have also been reported to yield a similar decrease in peak height^37^. In contrast to these works, the P K-edges in this work (Fig. 4d) exhibited a complete edge shift towards a higher energy level of electrodes harvested from OCV to ~2.91 V and then an edge shift back to a lower energy level from ~2.91 to 4.0 V, in addition to significant changes in the spectrum’s shape at the near edge. Only in the initial stage of charging did the P K-edge exhibited a slight increase in the near edge features and decrease in edge height (Fig. 4e) similar to the aforementioned literature. The spectrum taken at ~2.46 V already reveals a major change in shape, suggesting changes to the bonding environment of P have occurred. Subsequent sample measurements at higher voltages continued to produce pronounced changes to both the edge position and shape. The peak features at ~2151 and ~2167 eV might be related P_2_O_5_ present in the sample as shown in the comparison with commercial P_2_O_5_ experimental data (Supplementary Figure 6). As both peaks are consistently present among all the P K-edge spectrum, one might claim it is due to air contamination during measurement. However, the complete dominance of these higher energy peaks at ~3.17 V and its shift back towards lower energy levels for spectra taken at ~3.62 and ~4.0 V might indicate that this is not solely due to air contamination. Conversely, we believe the peak at ~3.17 V is related to the voltage and charging process of Li_2_S with LPS. Since chemical reactions between Li_3_PS_4_ and S_8_ have been reported literature, where the physical mixing of LPS with S_8_ have yielded Li polysulfidophosphates^38^. We propose that the higher energy levels observed from electrodes harvested at ~2.91 to ~3.62 V is due to some bonding of P with long-chained S species. With more sulfur species attached to each P atom, the electron cloud should shift away from P, subsequently increasing the excitation energy required for the P 1*s* core level. It should be noted that this is only our interpretation of our data and another chemical process could be occurring. Regardless of the true chemical interaction mechanism of LPS, even more interesting is that at the end of charge, that is, spectrum taken at 4.0 V, the XANES spectrum almost reverts completely back to the features of the sample taken at ~2.46 V (Fig. 4f). This suggests that the decomposed LPS performed a catalytic role. Because it does not revert to the same form as the spectrum taken at OCV or ~2.43 V, we believe the observed edge shifts toward a higher energy level for electrodes harvested from OCV to ~2.43 V could be an indication of an initial priming (“switching-on”) process of the LPS.

### Proposed mechanism

**Fig. 5:**
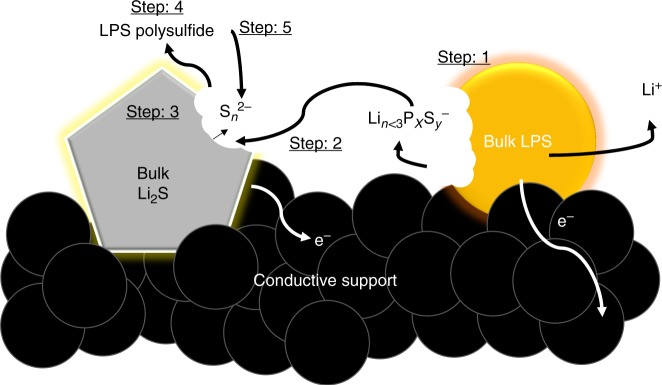
Schematic of proposed Li_2_S activation mechanism. Step 1: lithium thiophosphate (LPS) is delithiated generated soluble redox active P,S-based species. Step 2: These species diffuse to the surface of bulk Li_2_S, where it (Step 3) reacts via comproportionation. Step 4: The delithiated LPS form some of reversible compound with polysulfide. Step 5: The LPS-polysulfide compound is then used to further comproportionation with Li_2_S

Overall, the LPS initially delithiated, that is, “switched-on”, producing soluble redox active products and activating Li_2_S as shown in Step 1 of the schematic drawn in Fig. 5. The soluble redox-active products then migrated to the bulk crystalline Li_2_S (Step 2) where it helped generate polysulfide species (Step 3). This is also supported by the double activation peak shown in Fig. 2c. To follow, we believe the P from the delithiated LPS bonded with sulfur-based species (likely high ordered polysulfides, Step 4), stabilizing it for subsequent use in comproportionation reactions with the remaining Li_2_S particles (Step 5). When taken together with the differences in the EIS voltage profiles, we believe some of the formed delithiated LPS polysulfide is likely to offer a longer redox-active lifetime over the typical polysulfide species as there is no need for the reactivation of any species after each EIS data collection period. At higher voltages, the long-chained polysulfide species detach from the LPS via the oxidation of high-order polysulfide to elemental S_8_ (Fig. 3b at ~3.62 and 4.0 V), where the P K-edge begins to shift back to the lower energy level and finally back to the same edge energy level and features as the ~2.46 V spectrum at the end of charge (Fig. 3e). This indicates some form of catalytic role of the LPS generated species in the charging process of Li_2_S.

### Electrochemical performance

**Fig. 6:**
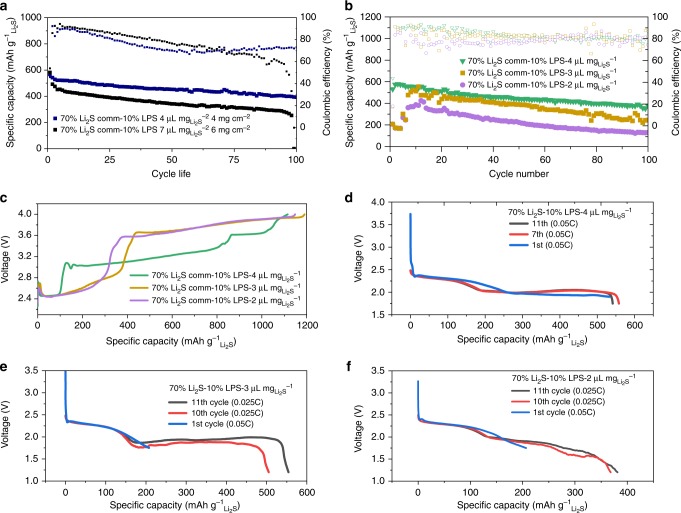
Electrochemical performance with lithium thiophosphate (LPS) added as an electrochemically “switched-on” redox mediator generator. **a** Cycle performance of 70% Li_2_S-10% LPS at 4 mg cm^−2^ ($4 \,{\rm{\mu}}{\mathrm{{L}}}\,{{\mathrm{mg}}^{-1}_{{{\mathrm{{Li}}}_{2}} {\mathrm{S}}}}$) and 6 mg cm^−2^ ($7\,{\rm{\mu}}{\mathrm{{L}}}\,{\mathrm{mg}}^{-1}_{{{\rm{{Li}}}_{2}}{\rm{S}}}$), **b** cycle performance and **c** charging voltage profile of 70% Li_2_S-10% LPS electrode at 1.5 ${\mathrm{{mg}}}_{{{\mathrm{{Li}}}_{2}}{\mathrm{{S}}}}$ at various electrolyte content (4–2 µL ${\mathrm{{mg}}}^{-1} {\hbox{)}}{\,}_{{{\rm{{Li}}}_{2}} {\rm{S}}}$, and **d**–**f** discharge voltage profile of 70% Li_2_S-10% LPS electrode at 1.5 ${\mathrm{{mg}}}^{-1}_{{{\rm{{Li}}}_{2}}{\rm{S}}}$ cm^−2^ at 4, 3, and 2 µL ${\mathrm{{mg}}}^{-1}_{{{\rm{{Li}}}_{2}}{\rm{S}}}$, respectively

Half-cell cycling performance of LPS containing electrodes exhibited reasonably good performance at low electrolyte ratio and high mass loadings especially for an electrode mainly based on only commercial bulk Li_2_S. Figure 6a shows the cycle stability of 70% Li_2_S–10% LPS electrode at various mass loading and Fig. 6b shows the cycle stability at various electrolyte content. With decreasing electrolyte content from 4 to $2\,{\rm{\mu}}{\mathrm{{L}}}\,{\mathrm{mg}}^{-1}_{{{\rm{{Li}}}_{2}}{\rm{S}}}$, an obvious and expected increase in charge overpotential is observed (Fig. 6c) with corresponding decreases in the discharge potential and capacity as shown in Fig. 6d–f. Cells at particularity low electrolyte content ($3--2\,{\rm{\mu}}{\mathrm{{L}}}\,{\mathrm{{mg}}}^{-1}_{{{\rm{{Li}}}_{2}}{\rm{S}}}$) required lower current density (0.025C) to achieve discharge. We would also like to emphasize the particularly high Li_2_S content in the electrode (70%) in comparison to other works of elaborate material structural design^39–41^. In contrast, pure Comm-Li_2_S cells even with 60% Li_2_S were only able to charge when activated past 4.0 V (0.025C) at such low electrolyte ratios as shown in Supplementary Figure 7a, b. Furthermore, as demonstrated in Fig. 1b, polysulfide-based activators demonstrate a similar problem, with significant increases in voltage and simultaneous decrease in charge capacity. This demonstrates the importance of solid-sourced redox mediator as a direction for future research. It is expected that with enhancements in the field of anode protection or separator/interlayer engineering^29,42^ beginning to gain traction, the performance can be significantly enhanced in subsequent work. Furthermore, future research into the direction of solid-sourced redox mediators based on other well-established solid-state electrolyte systems^43–45^ coupled with a deeper mechanistic understanding via in situ experiments^46,47^ will likely further increase the performance. Future improvements in capacity, reduction in first charge potential (related to the oxidative decomposition potential of the specific STSSE) while offering additional decoupling from the effects of low electrolyte content can be expected.

## Discussion

In summary, we have demonstrated here a method to leverage the commonly known oxidative decomposition of P, S-based solid-state electrolyte material. As an electrochemically “switched-on” redox mediator generator, we have introduced the first ever solid-sourced redox mediator for the activation of commercial bulk Li_2_S without the need for ball milling or high-energy treatment for electrode fabrication. By simply hand mortaring Li_2_S with LPS, there is substantial decrease in the charging potential. Results from EIS and X-ray adsorption spectroscopy indicate a disappearance in LPS material during the beginning of charge, followed by the formation of high resistance material likely to be sulfur. Furthermore, we believe there exist some bonding between the P and polysulfide species leading to the creation of redox-active species that have a longer life time during operation, allowing more opportunity for comproportionating with Li_2_S. Reasonable cycle performance was also demonstrated at low electrolyte content and reasonable Li_2_S mass loading with electrodes at 70 wt.% Li_2_S.

## Methods

### Materials

All chemicals and molecular sieves (0.3 nm, rod shapes of 1/16 in.) were purchased from Sigma-Aldrich. Molecular sieves were activated under vacuum at 120 °C for 48 h before use. Both  1,3 dioxolane (DOL) and dimethoxyethane (DME) was first dried with molecular sieves for 2 days. LiTFSI and LiNO_3_ was dried in a vacuum oven at ~ 120 °C overnight prior to electrolyte mixing. All other chemicals were used as received if not otherwise stated.

### Synthesis of materials

The synthesis of Li_3_PS_4_ (LPS) powder was conducted in a glove box with Ar atmosphere. High-purity precursors of Li_2_S (99.98%) (Sigma-Aldrich) and P_2_S_5_ (99%) (Sigma-Aldrich) were used as received, and anhydrous tetrahydrofuran (THF) solvent (99.9%) (Sigma-Aldrich) was pre-treated with molecular sieves to remove residual water before use. Li_2_S (0.244 g) and P_2_S_5_ (0.394 g) powders were mixed in the dried THF solution under stirring for 24 h. The prepared solution was pre-dried in the furnace for 24 h at 140 ℃ and then further dried in the vacuum oven for 24 h at 140 ℃. The β-Li_3_PS_4_ phase of our sample was confirmed by Raman spectroscopy (Supplementary Figure 8a)^48^, X-ray diffraction (Supplementary Figure 8b, c)^2^, and X-ray photoelectron spectroscopy (Supplementary Figure 8d, e)^49^. For testing lithium polysulfide as an Li_2_S activator, various amount of Li_2_S and S were mixed at a constant relative ratio (always forming Li_2_S_8_) at various absolute amounts in a solution consisting of DOL/DME at 1 (v/v) at ~50 °C overnight, forming a dark colored solution of Li_2_S_8_ at different concentrations.

### Electrochemical characterization

First polyvinylidene fluoride (PVDF, dried in vacuum oven at 60 °C) in *N*-methyl-2-pyrrolidone solution (dried with molecular sieves for 2 days and measured to be at ~5.1 ppm H_2_O by Karl Fischer titration) was mixed with solid LPS, Li_2_S, and finally Super C45 carbon black (C45, from Timical) with a final solid content of ~15%. Typical ratios of Li_2_S:LPS:C45:PVDF were 70:10:10:10 (10% LPS), 70:1:9:10 (1% LPS), and 60:10:20:10 (10% LPS with 60% Li_2_S). For the control sample without LPS, the slurry composition was 60:25:15 for Li_2_S:C45:PVDF, respectively. All slurries were hand mixed thoroughly in a mortar and pestle and blade casted to the desired thickness on an Al current collector. The electrode laminates were dried in a vacuum oven at 60 °C overnight and punched into 16 mm diameter disks. The entire electrode fabrication process was conducted inside of an Ar-filled glove box with H_2_O at <0.6 ppm and O_2_ <0.5 ppm. CR2032-type coin cells were used for all electrochemical measurements with a Li chip as the counter and reference electrode. For ex situ X-ray adsorption spectroscopy experiment, 0.5 LiNO_3_ in DME/DOL (1:1 v/v) was used as electrolyte to prevent signal convolution from the sulfur present in LiTFSI. For the LiPS baseline experiments, 30 µl of 1 M LiTFSI + 0.5 LiNO_3_ in DME/DOL (1:1 v/v) electrolyte was first injected onto the side anode and the separator, followed by the injection of 10 µl of electrolyte pre-blended with predetermined amount/concentration of LiPS directly onto the cathode. This was done to ensure the proper contact of polysulfide with Li_2_S and limit anode corrosion prior to first charge. For all other electrochemical test, 1 M LiTFSI + 0.5 M LiNO_3_ in DME/DOL (1:1 v/v) was used as the electrolyte. An 18 mm diameter 2320 Celgard membrane was used as the separator in all experiments. All galvanostatic cycling and rate performance tests were conducted with a Neware battery testing station. A 1400 CellTest System from Solartron was used to conduct cyclic voltammetry and EIS. EIS was conducted at 40 min constant current intervals, followed by a 5 min rest time prior to the EIS data collection.

### Physical and chemical characterization

X-ray adsorption spectroscopy experiments (operated under fluorescence mode) were conducted at the 9-BM of the Advanced Photon Source (APS) at Argonne National Laboratory (ANL). Cells were charged to their respective state of charge and quickly decrimped in an Ar-filled glove box. The harvested electrodes were dried without rinsing and sealed with a Kapton film-based sample holder to prevent air contamination. The X-ray adsorption spectroscopy experiment was conducted under He atmosphere. P_2_O_5_ and Na_2_S_2_O_3_ were used as the calibration sample for the P and S K-edge, respectively at the 9-BM of the APS at ANL. Using different segments of the same harvested electrodes, Raman spectroscopy was performed on a Renishaw In-Via Raman spectrometer with a 785 nm wavelength laser. X-ray diffraction was performed at the 11 ID-C (*λ* = 0.01173 nm) of the APS at ANL. Samples were sealed in Kapton tape to prevent air contamination. To prevent electrolyte leakage/evaporation, electrodes submerged in electrolyte were sealed in a coin cell with holes bored through the center of the top and bottom coin cell cap and covered with Kapton to allow for X-ray penetration.

## Supplementary information

Supplementary Information

## Data Availability

The data that support the findings of this study are available from the corresponding author upon reasonable request.
