# Supplementary material for: Electrochemically primed functional redox mediator generator from the decomposition of solid state electrolyte
Source: Nat Commun. 2019 Apr 23;10:1890. doi: 10.1038/s41467-019-09638-4 (PMC6478822; doi:10.1038/s41467-019-09638-4)
Supplement: Supplementary file 1 — Supplementary Information [file 41467_2019_9638_MOESM1_ESM.pdf]

Supplementary Information

Electrochemically Primed Functional Redox Mediator Generator from the Decomposition of Solid State Electrolyte

Li et al

Supplementary Figures:

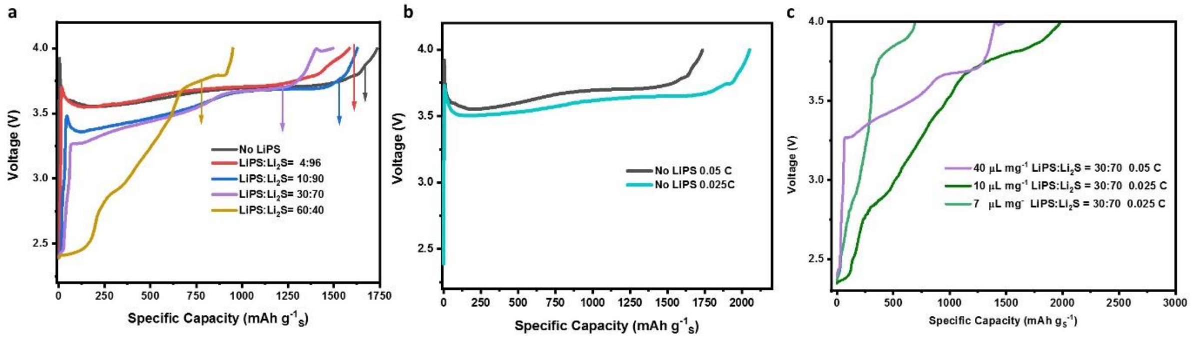

Supplementary Figure 1: **Effectiveness of Li_2S_8 as Li_2S activation agent at various operating condition** a) 1st charge voltage profile of 60% Comm-Li_2S loaded with varying amounts of Li_2S_8 in the electrolyte. Specific capacity is normalized to the total sulfur mass in both solid Li_2S and solvated Li_2S_8 mixture. Arrows indicate the theoretical delithiation capacity associated with each ratio of Li_2S_8:Li_2S. b) 1st charge voltage profile of pure 60% Comm-Li_2S electrode at 0.05 and 0.025 C. c) 1st charge voltage profile of commercial Li_2S at 30% Li_2S_8 and 40, 10 and 7 µL mg^{-1} electrolyte to equivalent S content at 0.05, 0.025 and 0.025C respectively.

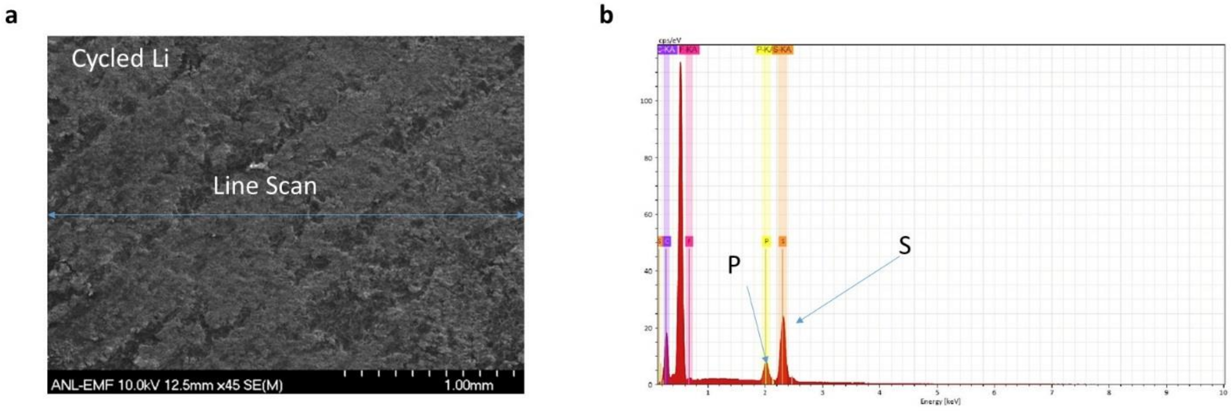

Supplementary Figure 2: Analysis of post cycling Li metal anode a) Scanning electron microscope image of cycled Li metal anode and b) the corresponding electron dispersive spectroscopy spectrum.

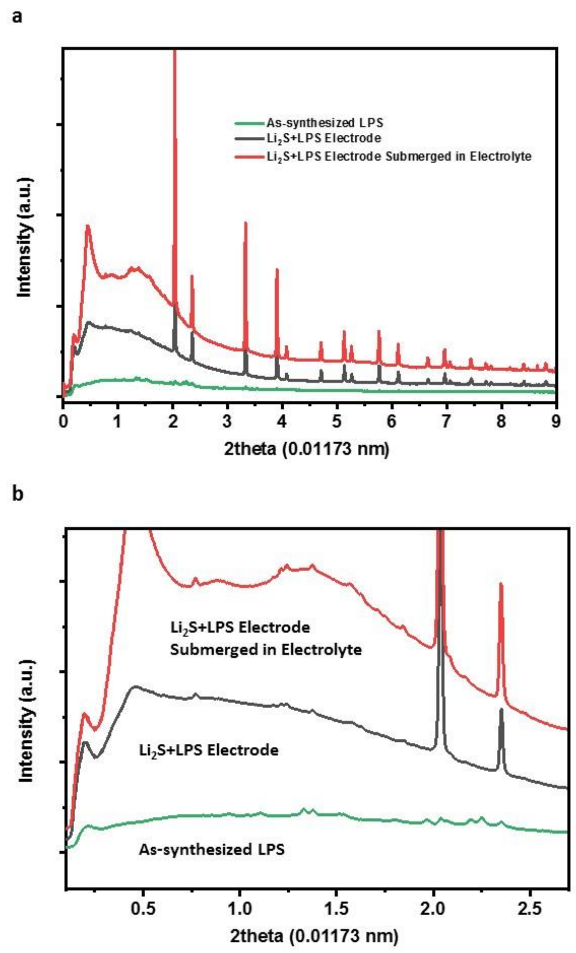

Supplementary Figure 3: Synchrotron high energy X-ray diffraction (λ=0.01173 nm) study of Li_3PS_4 at various stage of testing a) from 2θ = 1 to 9 and b) 2θ = 0.1 to 2.7 of as-synthesized LP, Li_2S+LPS electrode (scraped off from Al current collector) and Li_2S+Li_3PS_4 electrode scraped off from current collector and submerged in electrolyte for 2 hours and remained submerged during XRD testing.

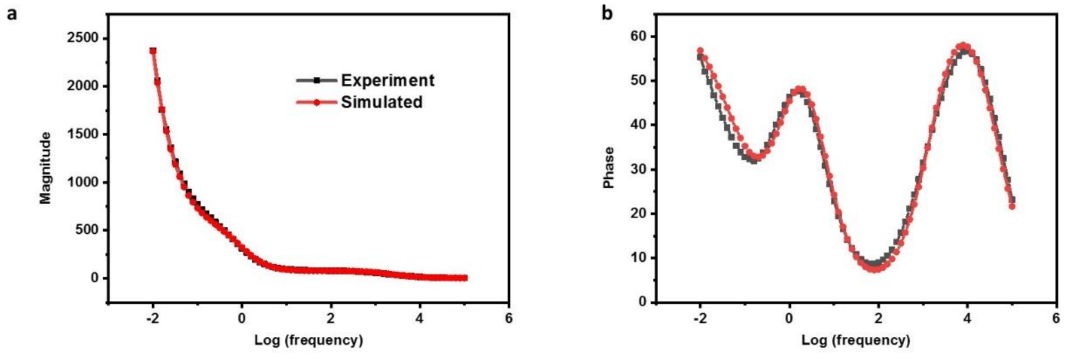

Supplementary Figure 4: Example of experimental and simulated electrochemical impedance spectroscopy plots a) magnitude and b) phase bode plot of Li_2S 60% commercial electrode with simulated and experimental data points in red and black respectively. c-d) Experimental EIS spectrums of Comm-Li_2S electrodes and f-h) of Comm-Li_2S electrodes blended with 10% Li_3PS_4 at different axis ranges. Unit of color legend is in volts.

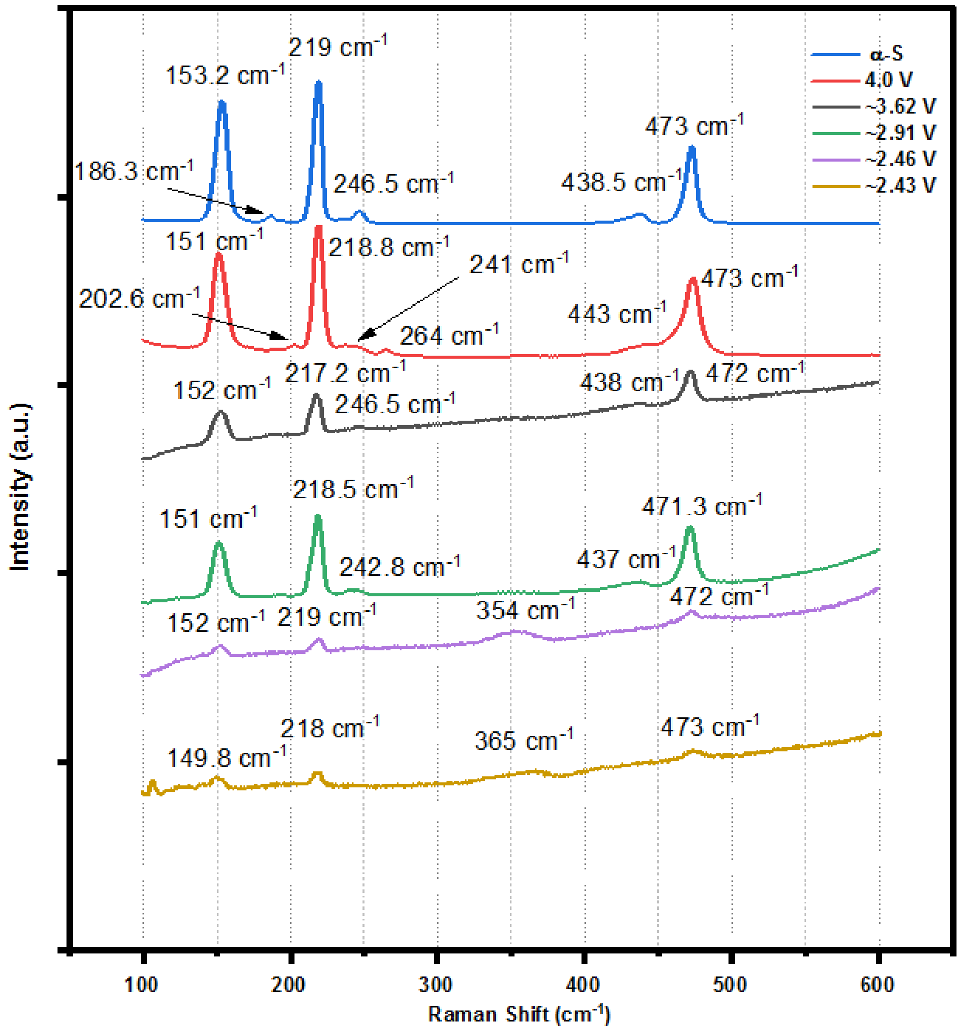

Supplementary Figure 5: Normalized Raman spectrum of Commercial S standard powder and Li_3PS_4+Li_2S electrodes charged to different voltages.

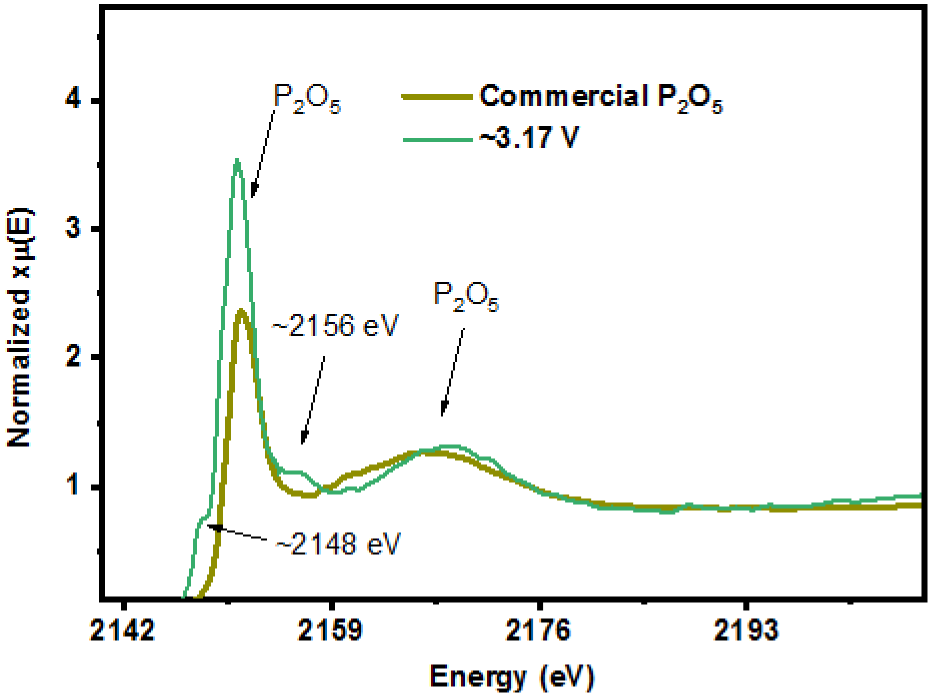

Supplementary Figure 6: Comparison of P K-edge spectrum of *ex situ* cell taken at ~2.91 V and the P_2O_5 standard sample.

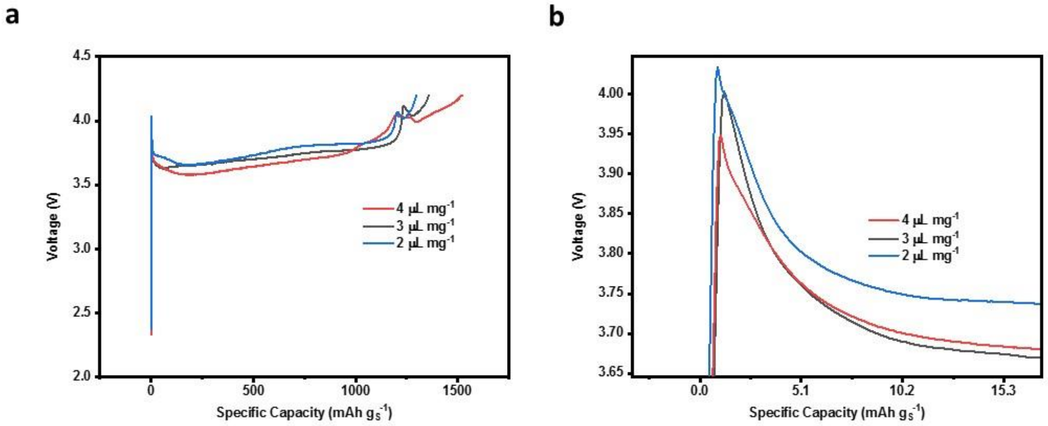

Supplementary Figure 7: Electrochemical properties of commercial Li_2S without Li_3PS_4 a) 1st charge voltage profile of 60% Comm-Li_2S at 4-2 µL mg^{-1} at 1.5 mg cm^{-2} and b) enlarged figure to show initial activation process.

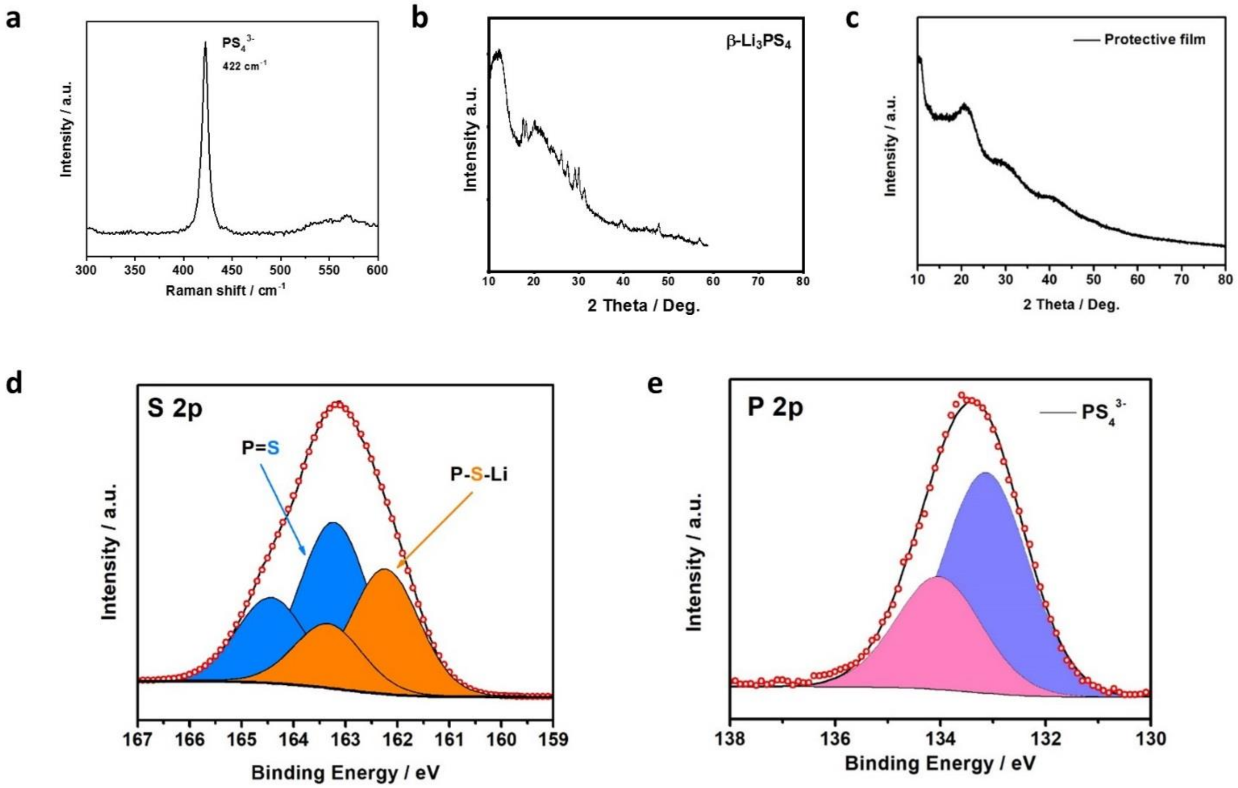

Supplementary Figure 8: Physical characterization of as-synthesized Li_3PS_4 a) Raman spectrum, b) X-ray diffraction of as-synthesized Li_3PS_4 protected with glass based air-tight sample holder, X-ray diffraction of the protective film without any samples, X-ray photoelectron spectroscopy at c) S 2p and d) P 2p of synthesized Li_3PS_4.

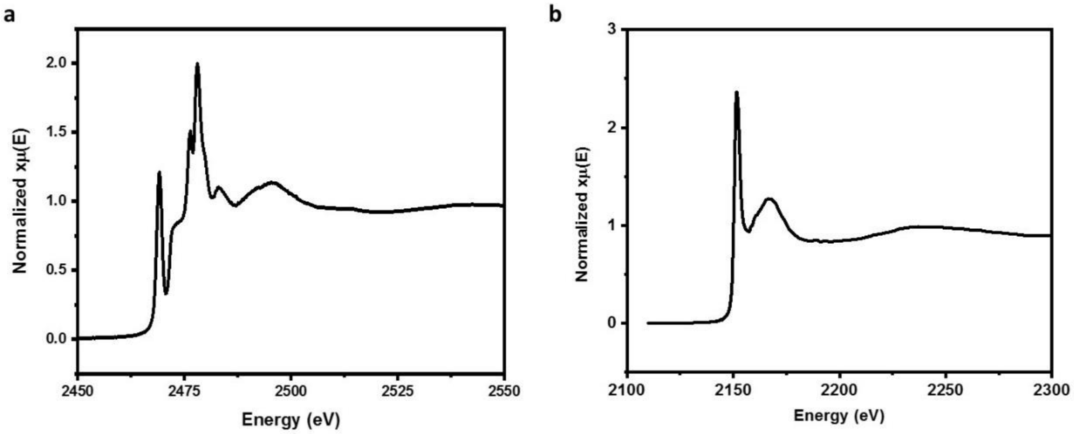

Supplementary Figure 9: X-ray adsorption near edge spectroscopy spectrum of calibrate samples. a) $Na_2S_2O_3$ at the S K-edge and b) P_2O_5 at the P K-edge.

Supplementary Table 1: Comparison between different Li_2S activation strategies.

Ref.	Li_2S Areal Loading (mg cm^{-2})	% Li_2S in electrode	1st overpotential peak	Current	1st Discharge Capacity (mAh g_S^{-1})
[1]	2	48	>3.0	200 mA/g	1014
[2]	0.8	N/A	>3.0	0.1C	1600
[3]	8	63%	3.8	0.05C	860
[4]	2	62%	2.7	0.1C	915
[5]	0.7-0.9	60	2.8	0.05C	915
[6]	1.2	N/A	2.4	0.1C	934.4
[7]	4	68-78	~3.4	0.1C	1350
[8]	2	60	n/a	0.05C	1109
This work	1.5	70	~2.7-2.8	0.05C	858

Supplementary References:

1. Peng, Y. et al. Constructing fast electron and ion conductive framework for Li_2S as advanced lithium sulfur battery. *Chem. Eng. J.* **346**, 57-64 (2018).
2. Klein, M. J., Dolocan, A., Zu, C. & Manthiram. A. An Effective Lithium Sulfide Encapsulation Strategy for Stable Lithium–Sulfur Batteries. *Adv. Energy Mater.* **7**, 1701122 (2017).
3. Chung, S.H., Han, P. Chang, C. H. & Manthiram. A. A Shell-Shaped Carbon Architecture with High-Loading Capability for Lithium Sulfide Cathodes. *Adv. Energy Mater.* **7**, 1700537 (2017).
4. Zhang, J., Shi, Y., Ding, Y., Peng, L., Zhang, W. & Yu, G. A Conductive Molecular Framework Derived Li_2S/N,P-Codoped Carbon Cathode for Advanced Lithium–Sulfur Batteries, *Adv. Energy Mater.* **7,** 1602876 (2017).
5. Hwa, Y., Zhao, J. & Cairns, E.J. Lithium Sulfide (Li_2S)/Graphene Oxide Nanospheres with Conformal Carbon Coating as a High-Rate, Long-Life Cathode for Li/S Cells, *Nano Lett.* **15**, 3479-3486 (2015).
6. Jiao, Z. et al. Core-shell $Li_2S@Li_3PS_4$ nanoparticles incorporated into graphene aerogel for lithium-sulfur batteries with low potential barrier and overpotential, *J. Power Sources.* **353**, 167-175 (2017)
7. Kohl, M., Brückner, J., Bauer, I., Althues, H. & Kaskel, S. Synthesis of highly electrochemically active Li_2S nanoparticles for lithium–sulfur-batteries, *J. Mater. Chem. A.* **3**, 16307-16312, (2015).
8. She, Z. W., Zhang, Q., Li, W., Zheng, G., Yao, H. & Cui, Y. Stable cycling of lithium sulfide cathodes through strong affinity with a bifunctional binder, *Chem. Sci.* **4**, 3673-3677 (2013).
